# Impact of heat on respiratory health: Age- and sex-specific risks in a nationwide Korean study (2014–2019)

**DOI:** 10.1007/s00484-026-03242-0

**Published:** 2026-06-17

**Authors:** Joonho Ahn, Jongmin Oh, Ho-Jang Kwon, Hyungryul Lim, Jonghyuk Choi, Sanghyuk Bae, Kyoung-Nam Kim, Mi-Ji Kim, Jong-Hun Kim, Youn-Hee Lim

**Affiliations:** 1 Department of Occupational and Environmental Medicine, Kangbuk Samsung Hospital, Sungkyunkwan University School of Medicine, Seoul, Republic of Korea; 2 Department of Environmental Medicine, Ewha Womans University College of Medicine, Seoul, Republic of Korea; 3 Institute of Ewha-SCL for Environmental Health (IESEH), College of Medicine, Ewha Womans University, Seoul, Republic of Korea; 4 Department of Human Systems Medicine, College of Medicine, Seoul National Unviersity, Seoul, Republic of Korea; 5 Integrated Major in Innovative Medical Science, Graduate School, Seoul National University, Seoul, Republic of Korea; 6 Department of Preventive Medicine, Dankook University College of Medicine, Cheonan, Republic of Korea; 7 Department of Preventive Medicine and Public Health, Ajou University School of Medicine, Suwon, Republic of Korea; 8 Department of Preventive Medicine, College of Medicine, The Catholic University of Korea, Seoul, Republic of Korea; 9 Department of Preventive Medicine, Yonsei University College of Medicine, Seoul, Republic of Korea; 10 Department of Preventive Medicine, Institute of Health Sciences, Gyeongsang National University College of Medicine, Jinju, Republic of Korea; 11 Department of Social and Preventive Medicine, Sungkyunkwan University School of Medicine, Suwon, Republic of Korea; 12 Section of Environmental Health, Department of Public Health, University of Copenhagen, Øster Farimagsgade 5, København K, 1353 Denmark

**Keywords:** Asthma, Chronic Obstructive Pulmonary Disease, Emergency department visits, Pneumonia, Respiratory Tract Infections, Temperature

## Abstract

Respiratory diseases pose a major global public health challenge. With climate change contributing to more frequent and intense heat events, understanding the effects of high temperatures on respiratory health is increasingly critical. However, existing evidence remains inconsistent. A nationwide time-series study was conducted to evaluate the association between daily maximum temperatures and emergency department (ED) visits for cause-specific respiratory diseases across 16 regions in South Korea during the warm season (April–September) from 2014 to 2019. Generalized additive models with quasi-Poisson regressions were applied, adjusting for long-term trends, humidity, day of the week, and air pollutants. Subgroup analyses were performed by age and sex. Each 1 °C increase in maximum temperature was associated with a significant increase in ED visits for total respiratory diseases (relative risk [RR] = 1.008; 95% confidence interval [CI]: 1.006–1.009). Stronger associations were observed for pneumonia (RR = 1.014; 95% CI: 1.012–1.016), COPD (RR = 1.010; 95% CI: 1.007–1.012), asthma (RR = 1.009; 95% CI: 1.007–1.011), and upper respiratory infections (RR = 1.005; 95% CI: 1.003–1.007). Males, children (< 15 years), and the elderly (≥ 65 years) were particularly vulnerable. High ambient temperatures are significantly associated with increased ED visits for respiratory diseases. These findings provide important evidence to guide governmental decision-making in developing heatwave preparedness policies, including targeted interventions, early warning systems, and healthcare resource planning for vulnerable populations.

**Supplementary Information:**

The online version contains supplementary material available at 10.1007/s00484-026-03242-0.

## Introduction

Respiratory diseases are a leading cause of global morbidity and mortality, contributing significantly to healthcare burdens and reduced quality of life (Viegi et al. 2020). Conditions such as asthma, chronic obstructive pulmonary disease (COPD), and respiratory infections are particularly sensitive to environmental factors (Vicedo-Cabrera et al. 2023).

Climate change has intensified the frequency and severity of extreme heat events, raising concerns about their impact on respiratory health (De Sario et al. 2013; Romanello et al. 2023). Elevated temperatures can aggravate respiratory conditions by increasing airway inflammation, altering immune responses, and intensifying exposure to pollutants such as ozone, which peaks during hot periods (Wang et al. 2026; Xu et al. 2023; Liu et al. 2025). However, existing studies have reported inconsistent associations between temperature and respiratory morbidity, likely due to geographic differences, population characteristics, and varying definitions of health outcomes (Zhang et al. 2019; Park et al. 2020; Lim et al. 2019; Song et al. 2018).

Some studies have shown that high temperatures increase ED visits or hospitalizations for respiratory illnesses (Reid et al. 2012; Sherbakov et al. 2018; van Loenhout et al. 2018; Michelozzi et al. 2009; Soneja et al. 2016; Song et al. 2018), while others have reported null associations (Phung et al. 2017; Park et al. 2020; Lim et al. 2019; Son et al. 2014; Smith et al. 2016; Ogbomo et al. 2017; Wang et al. 2012). These discrepancies highlight an urgent need to address the persistent inconsistencies in the global literature, particularly through comprehensive, cause-specific analyses that account for diverse geographic and demographic contexts. To bridge this research gap, the present study explicitly hypothesized that high ambient temperatures are significantly associated with increased ED visits for both infectious and non-infectious respiratory diseases in South Korea, with the magnitude of risk varying by disease subtype. This study aims to test this hypothesis by evaluating cause-specific associations and identifying vulnerable populations susceptible to heat-related respiratory risks.

## Materials and methods

### Study population

**Fig. 1:**
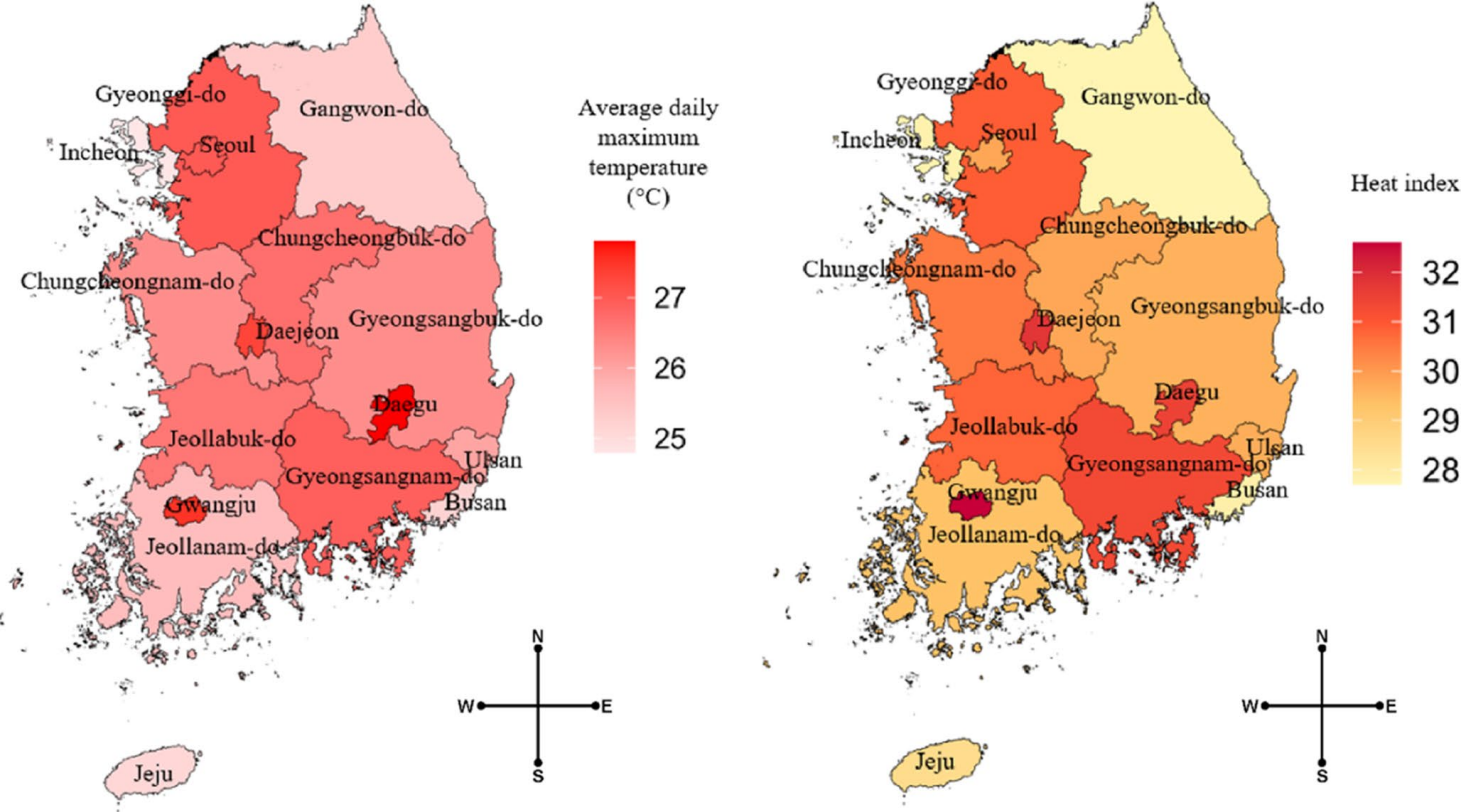
Distribution of average daily maximum temperature (°C) (left) and heat index (right) across 16 regions in South Korea during the warm season (April–September)

A nationwide time-series study was conducted to evaluate the association between daily maximum temperatures and emergency department (ED) visits for cause-specific respiratory diseases across 16 regions in South Korea during the warm season (April–September) from 2014 to 2019. The study included 16 regions in South Korea, comprising seven metropolitan cities (Seoul, Incheon, Daejeon, Daegu, Gwangju, Ulsan, and Busan) and nine provinces (Gyeonggi, Gangwon, Chungcheongbuk-do, Chungcheongnam-do, Gyeongsangbuk-do, Gyeongsangnam-do, Jeollabuk-do, Jeollanam-do, and Jeju) (Fig. 1).

### Health outcome variables

Health outcome data were obtained from the National Emergency Department Information System (NEDIS) database of South Korea. NEDIS was launched in 2003 by the Ministry of Health and Welfare and is currently managed by the National Emergency Medical Center of South Korea. As of 2019, the NEDIS database obtains information on ED admissions from 521 emergency institutions, including 119 local emergency centres and 402 local emergency institutes. NEDIS records all clinical and administrative data of patients who visit EDs nationwide, aiming to maintain ED quality and improve the emergency medical service system. With more than 98% of EDs participating in the system and an annual governmental review of the data, NEDIS is widely considered a trustworthy source of ED data. NEDIS data include individual characteristics (sex, age, and geographical region) and admission information (admission date and cause of disease with primary and secondary diagnosis codes).

ED visits for respiratory diseases were identified utilizing primary or secondary diagnosis codes in accordance with the International Classification of Diseases-10 (ICD-10), and the number of daily visitors was enumerated for each of the 16 regions in South Korea between 2014 and 2019. Among ED visits for respiratory diseases (J00-J99), four major cause-specific respiratory diseases, including acute upper respiratory infections (J00-J06), pneumonia (J09-J18), asthma (J45-J46), and COPD (J40-J44), were identified.

### Environmental exposure

Environmental exposure data were linked to ED visits according to patients’ residential region. The automated synoptic observing systems operated by the Korea Meteorological Administration were used to measure the daily maximum temperatures (°C) and relative humidity (%) for each region. Data were gathered from 98 observation stations during the warm season (April–September) between 2014 and 2019, excluding stations located on remote islands or mountains to preserve statistical integrity and prevent data bias, as well as those with less than 5 years of records. For regions with multiple observation stations, the daily mean of meteorological variables was computed from multiple stations within the region.

Moreover, for the robustness of the model, hourly average concentrations of particulate matter ≤ 10 μm in diameter (PM10), sulfur dioxide (SO2), nitrogen dioxide (NO2), carbon monoxide (CO), and ozone (O3) were acquired from AirKorea, which is managed by the National Institute of Environmental Research under the Ministry of Environment of South Korea. The measurement methods for PM10, SO2, NO2, CO, and O3 include beta-ray absorption, pulsed ultraviolet fluorescence, chemiluminescence, non-dispersive infrared, and ultraviolet photometry, respectively. Using the hourly data, the daily concentrations of PM10, SO2, NO2, CO, and O3 were computed in each city. Similar to meteorological data, region-specific daily mean levels of air pollution were computed.

### Statistical analysis

A two-stage time-series approach was used. In Stage 1, region-specific associations between daily maximum temperature and respiratory ED visits were estimated using quasi-Poisson generalized additive models (GAMs). To account for the delayed effects of temperature, cumulative moving average exposure windows were employed, ranging from same-day exposure (lag 0) to a six-day cumulative window (lag 0–5; the average of the visit day and the preceding five days). These windows were constructed by calculating the average temperature of the visit day and the preceding one to five days (e.g., lag 0–1, lag 0–2, …, lag 0–5), thereby capturing the sustained impact of heat exposure rather than the effect of a single-day lag. The model controlled for long-term trends (using smoothed spline functions with 4 degrees of freedom per year), daily mean humidity (%), and day of the week (DOW). Generalized Additive Models (GAM) were constructed as follows:

$$\begin{array}{l}{\mathrm{Y}}_\mathrm{t}\sim\mathrm{quasi}-\mathrm{Poisson}\left(\mathrm{E}\left(\mathrm{Y}\right)\right)\\\mathrm{log}\left(\mathrm{E}\left[\mathrm{Yt},\mathrm{k}\right]\right)\sim\alpha+\beta_1\times\mathrm{Tt},\mathrm{k}+\mathrm{s}\left(\mathrm{time}\right)+\mathrm{s}\left(\mathrm{daily}\;\mathrm{mean}\;\mathrm{humidity}\right)+\beta_2\times\mathrm{DOW}\end{array}$$

where E[Yt, k] represents the estimated daily number of emergency department visits on day t for a given cumulative lag window k; Tt, k denotes the cumulative moving average of the daily maximum temperature, ranging from same-day exposure (lag 0, where k = 0) to a six-day cumulative window (lag 0–5, where k = 5); and time is the calendar day with four *df* per year.

In Stage 2, the estimates of the associations for same-day exposure (lag 0) between temperature and respiratory diseases in 16 regions were pooled to calculate the national average relative risk (RR) of respiratory diseases per 1 °C increase in the maximum temperature in South Korea (Viechtbauer 2010). Lag 0 was selected as the primary representative index for this nationwide pooling to ensure consistency across the 16 regions and to capture the immediate physiological responses to heat.

GAM with nonparametric smoothing functions (penalized splines) was used to capture nonlinear relationships and estimate the association between the temperature at 0-day and respiratory diseases (Wood 2001). A subgroup analysis was performed by sex (male/female) and age group (< 15, 15–64, and ≥ 65 years) at 0-day.

In a sensitivity analysis, air pollutants (PM_10_, SO_2_, NO_2_, and O_3_) were additionally adjusted for in the GAM to explore the independent association of temperature and air pollutants with respiratory diseases (Ko and Kyung 2022). In another sensitivity analysis, the results of the main analysis were compared with the analysis using the heat index, which was introduced by Rothfusz (Ahn et al. 2023; Rothfusz and Headquarters 1990).

All statistical analyses were conducted using R software version 4.2.2 (The R Foundation for Statistical Computing, Vienna, Austria; 2022) with “*mgcv”* and “*metafor”(Wood* 2001*; Viechtbauer *2010).

## Results

Between 2014 and 2019, 3,695,723 cases of respiratory diseases were reported (Table 1). The number of ED visits was higher in males than in females. The 15–64 year age group had the highest incidence of respiratory diseases. Among the respiratory diseases, 2,322,542 cases were admitted to the hospital due to acute upper respiratory infections, 690,742 cases of pneumonia, 161,075 cases of asthma, and 237,586 cases of COPD from 2014 to 2019. The average ambient maximum temperature (°C) and the heat index during the warm season (April–September) are listed in Fig. 1. Among the 16 cities, Daegu had the highest average ambient maximum temperatures from 2014 to 2019 (Supplementary Table 1).

**Table 1:** Nationwide emergency department visits for cause-specific respiratory diseases during the warm season (April–September, 2014–2019) in South Korea

Diseases	Number of emergency visits					
	Overall	Sex		Age		
		Male	Female	< 15 years	15–64 years	≥ 65 years
Total respiratory diseases	3,695,723	1,981,244	1,714,479	1,414,730	1,572,399	708,594
Acute upper respiratory infections	2,322,542	1,194,299	1,128,243	1,093,584	1,109,634	119,324
Pneumonia	690,742	388,623	302,119	179,996	175,630	335,116
Asthma	161,075	83,101	77,974	33,161	61,998	65,916
COPD	237,586	154,608	82,978	28,351	75,514	133,721

*COPD* chronic obstructive pulmonary disease

**Fig. 2:**
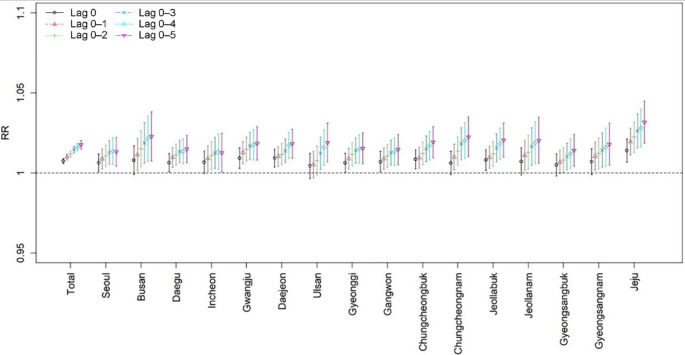
Relationship between daily maximum temperature and number of total respiratory diseases by lag days and regions. Abbreviations: RR = relative risk. *Notes*. Lag 0 represents same-day exposure, and lag 0–k represents the cumulative moving average of daily maximum temperature from day 0 to day k

Across most regions, higher temperatures were consistently associated with increased respiratory ED visits, particularly for pneumonia and COPD, although the magnitude varied by region and lag structure. Figure 2 shows the region-specific RRs of total respiratory diseases associated with a 1 °C increase in the maximum temperature in the 16 regions stratified by lag days. The pooled effect showed a significant increase in the RR of total respiratory diseases at different lags and moving averages: at lag0, the RR was 1.008 (95% confidence interval [CI] 1.006–1.009); at the 1-day moving average, the RR was 1.010 (95% CI 1.008–1.012); at the 2-day moving average, the RR was 1.012 (95% CI 1.010–1.014); at the 3-day moving average, the RR was 1.015 (95% CI 1.012–1.017); at the 4-day moving average, the RR was 1.016 (95% CI 1.014–1.019); and at the 5-day moving average, the RR was 1.018 (95% CI 1.015–1.020). Among the 16 regions, regions located in the southern part of South Korea, including Busan, Ulsan, Jeollanam-do, Gyeongsangbuk-do, and Gyeongsangnam-do, and two north-western regions of South Korea (Chungcheongnam-do, Incheon) did not show any significant associations on certain days.

Supplementary Fig. 1 shows the combined RRs of specific respiratory diseases, such as acute upper respiratory infections, pneumonia, asthma, and COPD, associated with increased maximum temperature across all lag days in the 16 regions. For example, the RR was greatest in pneumonia (1.014 [95% CI 1.012–1.016]) followed by COPD (1.010 [95% CI 1.007–1.012]), asthma (1.009 [95% CI 1.007–1.011]), and acute upper respiratory infections (1.005 [95% CI 1.003–1.007]) per 1 °C increase in maximum temperature at lag 0 (Table 2).

**Table 2:** Associations between daily maximum temperature at lag0 and emergency department visits due to cause-specific respiratory diseases (per 1 °C increase in daily maximum temperature)

Outcome variables	Pooled effect size (RR, per 1 °C increase)
Total respiratory diseases	1.008 (1.006–1.009)
Acute upper respiratory infections	1.005 (1.003–1.007)
Pneumonia	1.014 (1.012–1.016)
Asthma	1.009 (1.007–1.011)
COPD	1.010 (1.007–1.012)

*COPD* chronic obstructive pulmonary disease, *RR* relative risk

When air pollutants were controlled for in the model, the association with temperature did not change (Supplementary Figs. 2 and 3). Similarly, the heat index, a proxy of apparent temperature, also showed results similar to those of the main analysis (Supplementary Figs. 4 and 5).

**Fig. 3:**
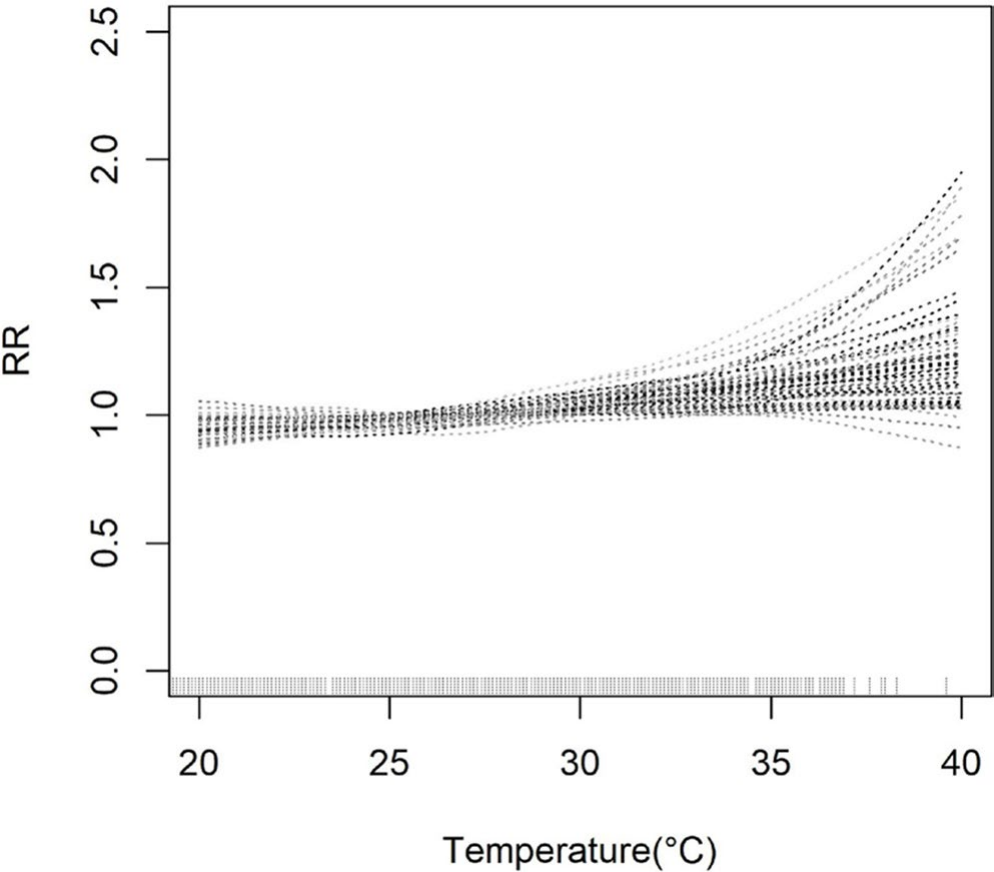
Nonlinear relationship between daily maximum temperatures and total respiratory diseases across 16 different regions in South Korea at lag0

**Fig. 4:**
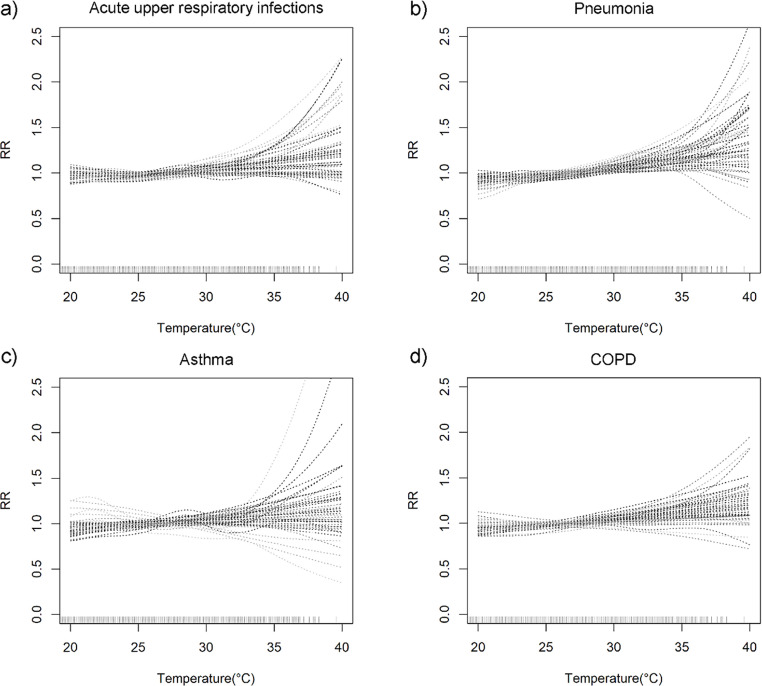
Nonlinear relationship between daily maximum temperatures and specific respiratory diseases (acute upper respiratory infections, pneumonia, asthma, and COPD) across 16 different regions in South Korea at lag0

Overall, all regions showed a generally increasing exposure–response relationship between temperature and respiratory diseases. Figure 3 depicts the positive nonlinear relationship between daily maximum temperatures at lag 0 and total respiratory diseases across the 16 regions. Similarly, Fig. 4 illustrates the region-specific positive nonlinear relationship between temperatures and specific respiratory diseases such as acute upper respiratory infections, pneumonia, asthma, and COPD in these 16 regions. Overall, the risk of respiratory diseases in all 16 regions showed an increasing trend with rising temperatures, suggesting a consistent rise in risk without distinct thresholds in most areas. The exposure-response functions showed similar patterns after adjusting for air pollutants in the model (Supplementary Figs. 6 and 7) and using heat index (Supplementary Figs. 8 and 9).

**Fig. 5:**
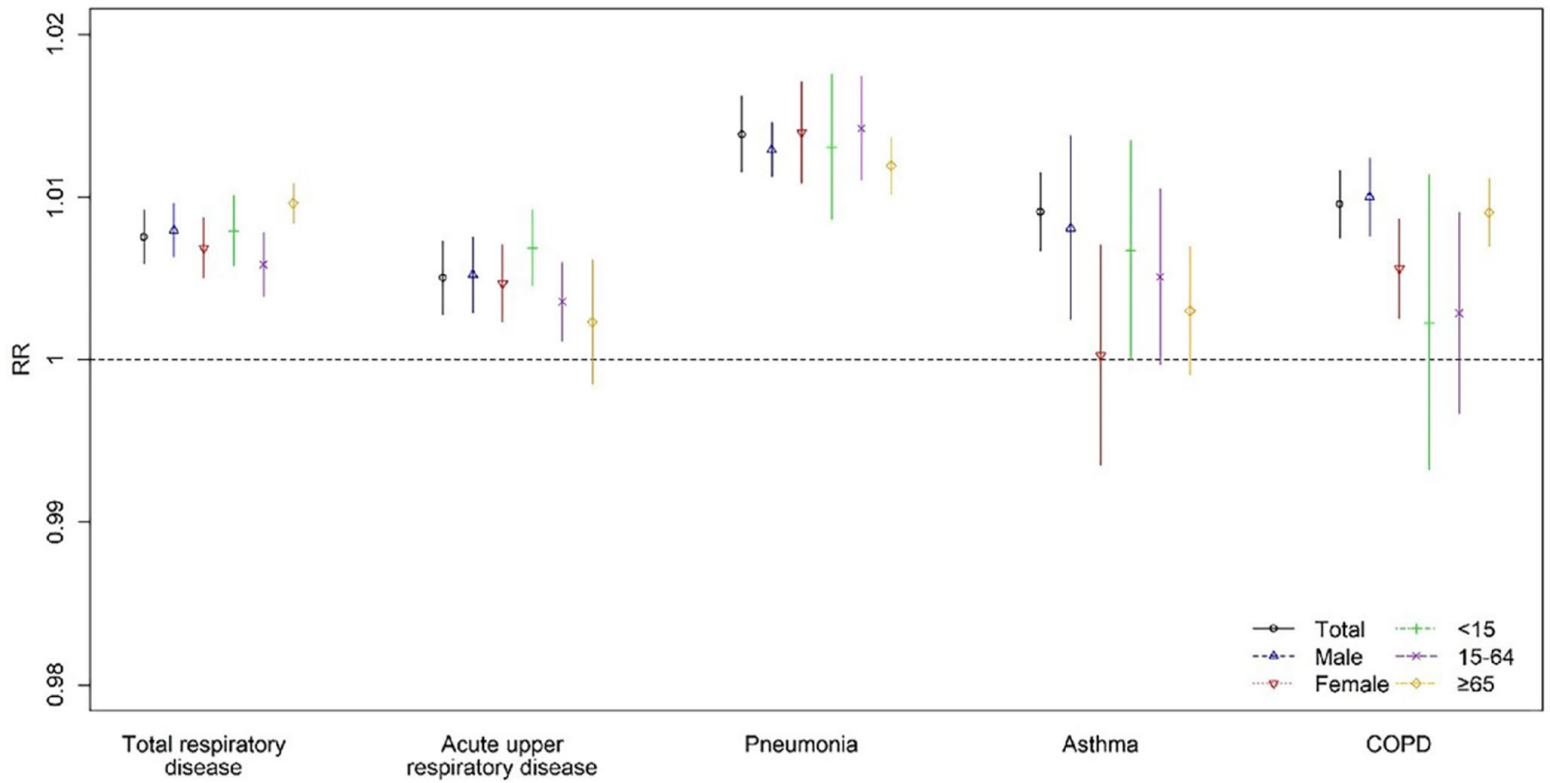
Subgroup analyses of the association between daily maximum temperature and specific respiratory diseases (acute upper respiratory infections, pneumonia, asthma, and COPD) stratified by sex and age

Populations susceptible to the adverse effects of ambient temperature for ED visits were identified at lag0. The associations between asthma, COPD, and ambient temperature were stronger in males than in females (Fig. 5). Most of these findings were consistent after controlling for air pollutants or using heat index (Supplementary Fig. 10 and Supplementary Fig. 11). In terms of age group differences in respiratory diseases due to temperature, the association with COPD was the greatest in the group aged ≥ 65 years (Fig. 5), whereas the association with acute upper respiratory infections was the greatest in children under 15 years old. In the sensitivity analyses, these age groups showed similar patterns of susceptibility to the association of temperature with overall respiratory disease outcomes (Supplementary Fig. 10 and Supplementary Fig. 11).

## Discussion

The nationwide analysis demonstrates a significant association between high ambient temperatures and increased ED visits for both total and specific respiratory diseases, including acute upper respiratory infections, pneumonia, asthma, and COPD. The effects were particularly pronounced among males, children, and older adults.

These findings remain robust after adjusting for air pollutants and applying alternative temperature indices. As global temperatures continue to rise, these results underscore the importance of integrating heat-related respiratory health risks into public health planning. Strategies such as heatwave preparedness programs, especially for vulnerable groups, are essential.

Previous studies on the impact of high temperatures on total respiratory disease have shown considerable variation (Reid et al. 2012; Sherbakov et al. 2018; van Loenhout et al. 2018; Arbuthnott and Hajat 2017; Basagaña et al. 2011; Zhang et al. 2019; Park et al. 2020; Lim et al. 2019; Son et al. 2012, 2014; Michelozzi et al. 2009; Soneja et al. 2016; Smith et al. 2016; Ogbomo et al. 2017; Vaneckova and Bambrick 2013; Wilson et al. 2013; Song et al. 2018; Wang et al. 2012; Braga et al. 2002; Fuhrmann et al. 2016), with some reporting a significant increase in respiratory diseases (Reid et al. 2012; Sherbakov et al. 2018; van Loenhout et al. 2018; Zhang et al. 2019) while others report no such association (Phung et al. 2017; Son et al. 2012, 2014; Lim et al. 2019; Smith et al. 2016; Ogbomo et al. 2017; De Sario et al. 2013). This pattern was also observed when examining infectious and non-infectious respiratory diseases. In some studies, the risk of infectious respiratory diseases (e.g., acute upper respiratory infections and pneumonia) was significantly increased with increasing temperature (Zhang et al. 2019; Wilson et al. 2013), whereas other studies reported no significant increase (Basagaña et al. 2011; Park et al. 2020; Wang et al. 2012; Braga et al. 2002). Similarly, for non-infectious respiratory diseases (e.g., asthma and COPD), some studies reported significant risk increases (Basagaña et al. 2011; Soneja et al. 2016; Wilson et al. 2013; Braga et al. 2002) and others reported no significant associations (Zhang et al. 2019; Vaneckova and Bambrick 2013).

Although a clear explanation for these mixed results is not available, it can be postulated that not only specific respiratory diseases but also the admission types of respiratory diseases play an important role in association studies. For example, most studies using ED visits as the outcome variable observed a significant increase in respiratory risk (van Loenhout et al. 2018; Song et al. 2018; Fuhrmann et al. 2016) in accordance with the present findings, whereas studies using more severe outcomes such as hospitalization or mortality mostly reported non-significant associations (Phung et al. 2017; Son et al. 2012, 2014; Park et al. 2020; Lim et al. 2019; Ogbomo et al. 2017). Therefore, further studies are needed to investigate the different associations by severity or prognosis status of the diseases associated with exposure to ambient temperature.

The mechanisms underlying the increased risk of respiratory diseases attributable to high temperatures remain unclear and may limit the interpretation of these findings. High temperatures may contribute to the formation of air pollutants that exacerbate respiratory conditions (Soneja et al. 2016). High temperatures contribute to the formation of atmospheric pollutants such as ground-level ozone, which can trigger respiratory inflammation and exacerbate respiratory diseases. However, even after adjusting for air pollution in the sensitivity analysis, robust results were observed, indicating the need for further consideration of additional mechanisms. In the case of infectious respiratory diseases, an experimental study showed that mice exposed to heat stress exhibit a decrease in respiratory immunity, making them more susceptible to highly pathogenic respiratory viruses (Jin et al. 2011). For non-infectious respiratory diseases, acute exacerbations are related to airway and systemic inflammation and accompanying cardiovascular diseases (Bathoorn et al. 2008). During hot weather, patients with these diseases may experience hyperventilation, which can exacerbate their condition (Michelozzi et al. 2009). Additionally, under extreme heat conditions, acute lung inflammation and injury may occur because of heatstroke, leading to physiological changes that can result in severe respiratory distress syndrome (Hsi-Hsing et al. 2010). Further research is required to better understand the relationship between high temperatures and respiratory diseases.

In particular, this study performed a multifaceted analysis by examining the associations for specific respiratory subtypes in relation to demographic factors such as sex and age, providing a more detailed perspective on population vulnerability. In the present study, a subgroup analysis by sex showed that the risks of asthma and COPD were higher in males than in females. Previous studies have yielded inconsistent results, with some showing similar effect sizes for both sexes (Zhang et al. 2019). Some reporting a higher risk in females (Son et al. 2012), and others indicating a higher risk in males (Soneja et al. 2016). Differences in risk between the sexes may be attributed not only to physiological factors, such as sex-related thermoregulatory differences, but also to social factors, such as gender roles (Fruttero et al. 2024; Doyal 2003). The variation in the study results could be due to a combination of these factors, suggesting the need for further research to account for these aspects. The age-stratified analysis revealed that individuals aged ≥ 65 years exhibited the strongest association with COPD, while children under 15 years were most affected by acute respiratory infections. Increased vulnerability among children and older people is generally attributed to their immature or reduced thermoregulatory abilities and increased susceptibility to respiratory infections (Schapiro et al. 2024; Núñez-Rodríguez et al. 2025; Li et al. 2015). However, the associations specific to age varied among studies (Michelozzi et al. 2009; Soneja et al. 2016; Song et al. 2018). It can be postulated that the discrepancy may arise from differences in healthcare systems and social support in the studied countries, making the drawing consistent conclusions challenging (Michelozzi et al. 2009). Nevertheless, universal health insurance and high healthcare accessibility in South Korea ensure the systematic capture of clinical events. The NEDIS database, covering more than 98% of emergency departments nationwide, provides a highly sensitive platform for detecting heat-related respiratory risks (Kim et al. 2024). This centralized system likely enhances the sensitivity of our results compared to studies in regions with fragmented data or significant healthcare barriers. Additionally, differences in indoor and outdoor activity times depending on age group can further complicate the interpretation of the relationship between high temperatures and health impacts (Kim et al. 2020).

This study had several strengths. First, to the best of the authors` knowledge, this is the first study in South Korea to examine the relationship between high temperatures and respiratory diseases on a national scale using ED visit data. Second, the results remained robust even after adjusting for air pollution and using different temperature indices as shown in sensitivity analyses. Third, the populations susceptible to the adverse effects of temperature for cause-specific ED visits were identified.

Fourth, beyond overall respiratory diseases, this study conducted a granular analysis of four specific respiratory outcomes by distinguishing infectious diseases (pneumonia and acute upper respiratory infections) from non-infectious diseases (asthma and COPD). In the international literature, findings on the association between high temperatures and respiratory diseases have been highly inconsistent, even when similar analytical frameworks have been applied, with mixed results reported for both infectious and non-infectious outcomes. These discrepancies have been attributed to differences in outcome definitions, disease severity, admission types, and heterogeneity in population vulnerability across settings. By jointly examining these four major respiratory subtypes in conjunction with age- and sex-specific vulnerabilities using a uniform methodological framework and emergency department visit data, the present study provides a coherent explanation for the heterogeneous findings reported in previous international studies. This disease-specific, comparative approach offers added value beyond age- and sex-stratified estimates and contributes to a more nuanced understanding of heat-related respiratory risks in the global context.

This study also had some limitations. First, respiratory diseases were identified using the ICD-10 codes from emergency department data. These codes may not provide an accurate representation of clinical details, which could result in potential misdiagnoses. Moreover, the exclusive use of emergency department records may introduce selection bias by excluding milder cases and overrepresenting populations with better access to healthcare. Second, our study was conducted in 16 regions, which limits the spatial resolution. A study with a high spatial resolution of exposure and health outcomes would have been possible if the research had been conducted at a smaller city or county level. This limitation includes the absence of intra-regional variability analysis, which may overlook differences between urban and rural areas or among neighborhoods with distinct socioeconomic and environmental profiles. However, there are issues with smaller area units, such as no cause-specific cases in less populated areas (Kim et al. 2020). To address these constraints, future studies should improve spatial resolution using high-resolution exposure data, such as satellite-based estimates, microclimate models, or individual-level sensors, to better capture spatial variability and reduce exposure misclassification. Third, the estimation of heat exposure based on regional averages does not account for individual-level factors such as time spent outdoors, housing conditions, or access to cooling systems. Fourth, susceptibility factors were primarily limited to age and sex. Other critical influences, including comorbidities, socioeconomic status, and behavioral or cultural factors such as daily routines and heat avoidance practices, were not fully considered (Shannon et al. 2025). Lastly, the generalizability of these findings may be limited to the South Korean context, as demographic, climatic, and healthcare system characteristics differ in other regions.

## Conclusion

The present nationwide study demonstrated a significant association between high ambient temperatures and increased emergency department visits for total and cause-specific respiratory diseases, including acute upper respiratory infections, pneumonia, asthma, and COPD. Notably, males, children, and the elderly exhibited heightened susceptibility to these effects. These findings remained robust after adjusting for air pollution and using alternative temperature indices.

Given the ongoing rise in ambient temperatures and the expected increase in extreme heat events due to climate change, the results provide critical evidence for developing targeted prevention strategies. Public health authorities should prioritize heatwave preparedness programs, especially for vulnerable groups. Future research should explore additional vulnerability factors, such as pre-existing conditions, socioeconomic status, and behavioral characteristics, and aim to improve the spatial resolution of exposure and health outcome data to better capture localized patterns and disparities, thereby informing more targeted public health interventions.

## Supplementary Information

Below is the link to the electronic supplementary material.

Supplementary Material 1

## Data Availability

The individual-level datasets analyzed during the current study are not publicly available due to restrictions under the Personal Information Protection Act. However, aggregated daily data are available from the corresponding author upon reasonable request.
