# Supplementary material for: Impact of heat on respiratory health: Age- and sex-specific risks in a nationwide Korean study (2014–2019)
Source: Int J Biometeorol. 2026 Jun 17;70(7):191. doi: 10.1007/s00484-026-03242-0 (PMC13275515; doi:10.1007/s00484-026-03242-0)
Supplement: Supplementary file 1 — Supplementary Material 1 [file 484_2026_3242_MOESM1_ESM.docx]

**Supplementary Material**

**Impact of Heat on Respiratory Health: Age- and Sex-Specific Risks in a Nationwide Korean Study (2014–2019)**

**Table of Contents**

**Supplementary Figures**

[Supplementary Fig. 1 Relationship between daily maximum temperature and the number of specific respiratory diseases (acute upper respiratory infections, pneumonia, asthma, and COPD) by lag days and regions 3](#_Toc191995810)

[Supplementary Fig. 2 Relationship between daily maximum temperature and the number of total respiratory diseases by lag days and regions, considering the first sensitivity analysis controlling for air pollutants (PM10, SO2, NO2, and O3) 4](#_Toc191995810)

[Supplementary Fig. 3 Relationship between daily maximum temperature and the number of specific respiratory diseases (acute upper respiratory infections, pneumonia, asthma, and COPD) by lag days and regions, considering the first sensitivity analysis controlling for air pollutants (PM_10_, SO_2_, NO_2_, and O_3_) 5](#_Toc191995811)

[Supplementary Fig. 4 Relationship between daily maximum temperature and number of total respiratory diseases by lag days and regions, considering the second sensitivity analysis using the heat index instead of daily maximum temperature 6](#_Toc191995812)

[Supplementary Fig. 5 Relationship between daily maximum temperature and number of specific respiratory diseases (acute upper respiratory infections, pneumonia, asthma, and COPD) by lag days and regions, considering the second sensitivity analysis using heat index instead of daily maximum temperature 7](#_Toc191995813)

[Supplementary Fig. 6 Nonlinear relationship between daily maximum temperatures and total respiratory diseases across 16 different regions in South Korea at lag0, employing a time-series model that controlled for air pollutants (PM_10_, SO_2_, NO_2_, and O_3_) by including them as covariates in the first sensitivity analysis 8](#_Toc191995814)

[Supplementary Fig. 7 A nonlinear relationship observed between daily maximum temperatures and specific respiratory diseases (acute upper respiratory infections, pneumonia, asthma, and COPD) across 16 different regions in South Korea, after controlling for air pollutants (PM_10_, SO_2_, NO_2_, and O_3_) in the first sensitivity analysis 9](#_Toc191995815)

[Supplementary Fig. 8 Nonlinear relationship between daily maximum temperatures and total respiratory diseases across 16 different regions in South Korea at lag day 0, using the heat index instead of the daily maximum temperature in the second sensitivity analysis 10](#_Toc191995816)

[Supplementary Fig. 9 A nonlinear relationship between daily maximum temperatures and specific respiratory diseases (acute upper respiratory infections, pneumonia, asthma, and COPD) across 16 different regions in South Korea at lag0, using the heat index instead of the daily maximum temperature in the second sensitivity analysis 11](#_Toc191995817)

[Supplementary Fig. 10 Subgroup analyses of the associations between daily maximum temperature and specific respiratory diseases (acute upper respiratory infections, pneumonia, asthma, and COPD) stratified by sex and age groups, controlling for air pollutants (PM_10_, SO_2_, NO_2_, and O_3_) in the first sensitivity analysis 12](#_Toc191995818)

[Supplementary Fig. 11 Subgroup analyses of the associations between daily maximum temperature and specific respiratory diseases (acute upper respiratory infections, pneumonia, asthma, and COPD) stratified by sex and age groups using the heat index instead of daily maximum temperature in the second sensitivity analysis 13](#_Toc191995819)

**Supplementary Tables**

[Supplementary Table 1. City- and province-level population, emergency department visits, and respiratory diseases during the warm season 14](#_Toc191996219)

Supplementary Fig. 1 Relationship between daily maximum temperature and the number of specific respiratory diseases (acute upper respiratory infections, pneumonia, asthma, and COPD) by lag days and regions


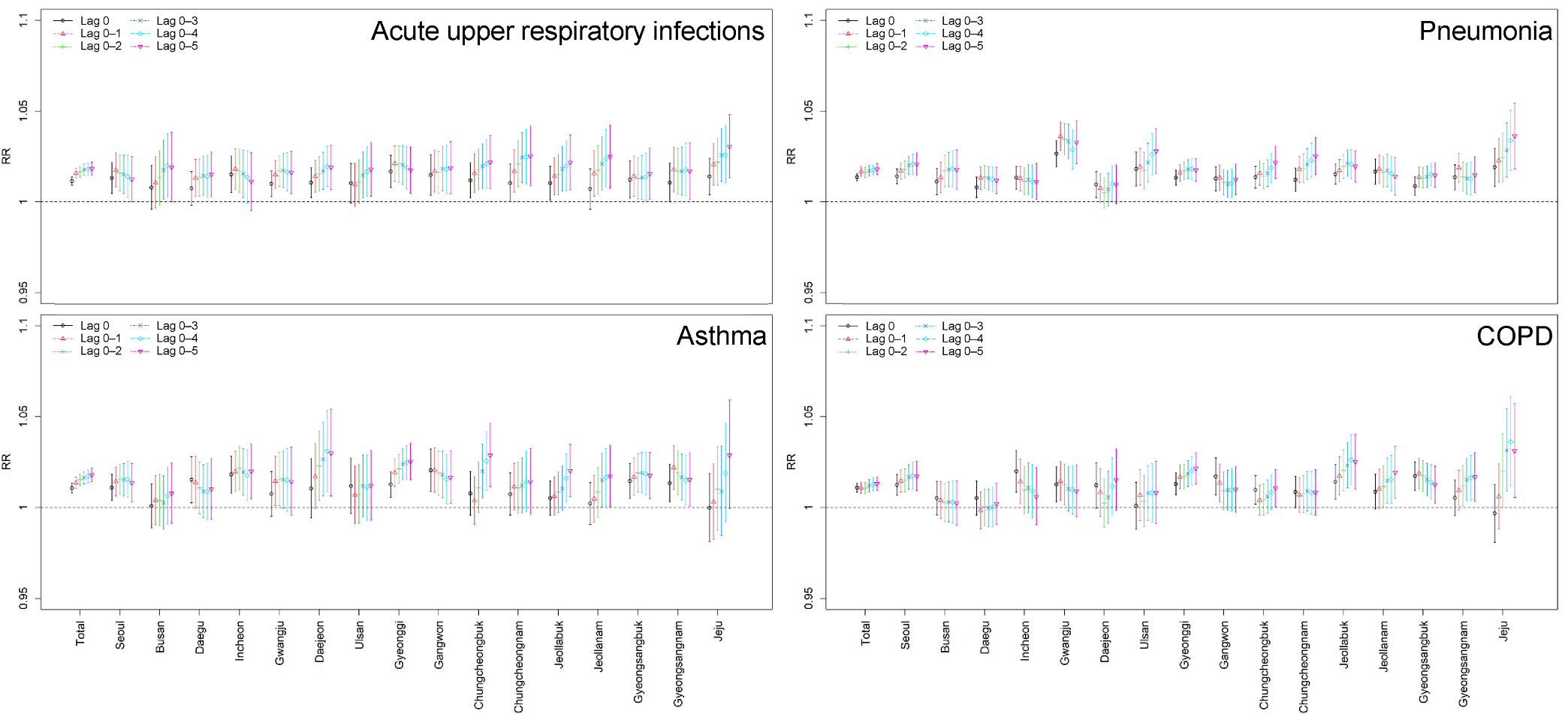


* COPD, chronic obstructive pulmonary disease; RR, relative risk per 1°C increase in daily maximum temperature. Lag 0 represents same-day exposure, and lag 0–k represents the cumulative moving average of daily maximum temperature from day 0 to day k.

**Supplementary** Fig. 2 Relationship between daily maximum temperature and the number of total respiratory diseases by lag days and regions, considering the first sensitivity analysis controlling for air pollutants (PM_10_, SO_2_, NO_2_, and O_3_)


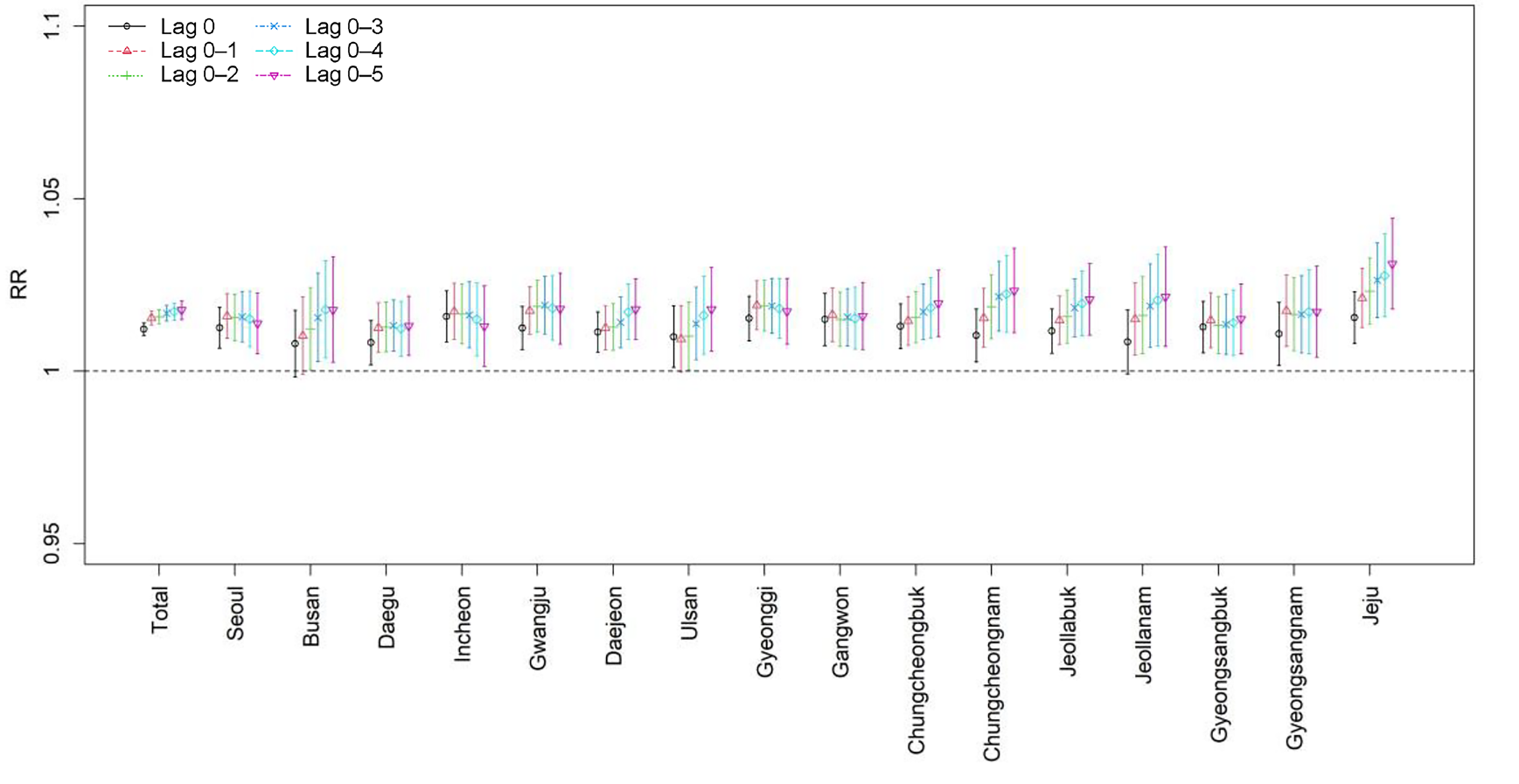


* PM_10_, particulate matter ≤10 µm in diameter; RR, relative risk per 1°C increase in daily maximum temperature. Lag 0 represents same-day exposure, and lag 0–k represents the cumulative moving average of daily maximum temperature from day 0 to day k.

Supplementary Fig. 3 Relationship between daily maximum temperature and the number of specific respiratory diseases (acute upper respiratory infections, pneumonia, asthma, and COPD) by lag days and regions, considering the first sensitivity analysis controlling for air pollutants (PM_10_, SO_2_, NO_2_, and O_3_)


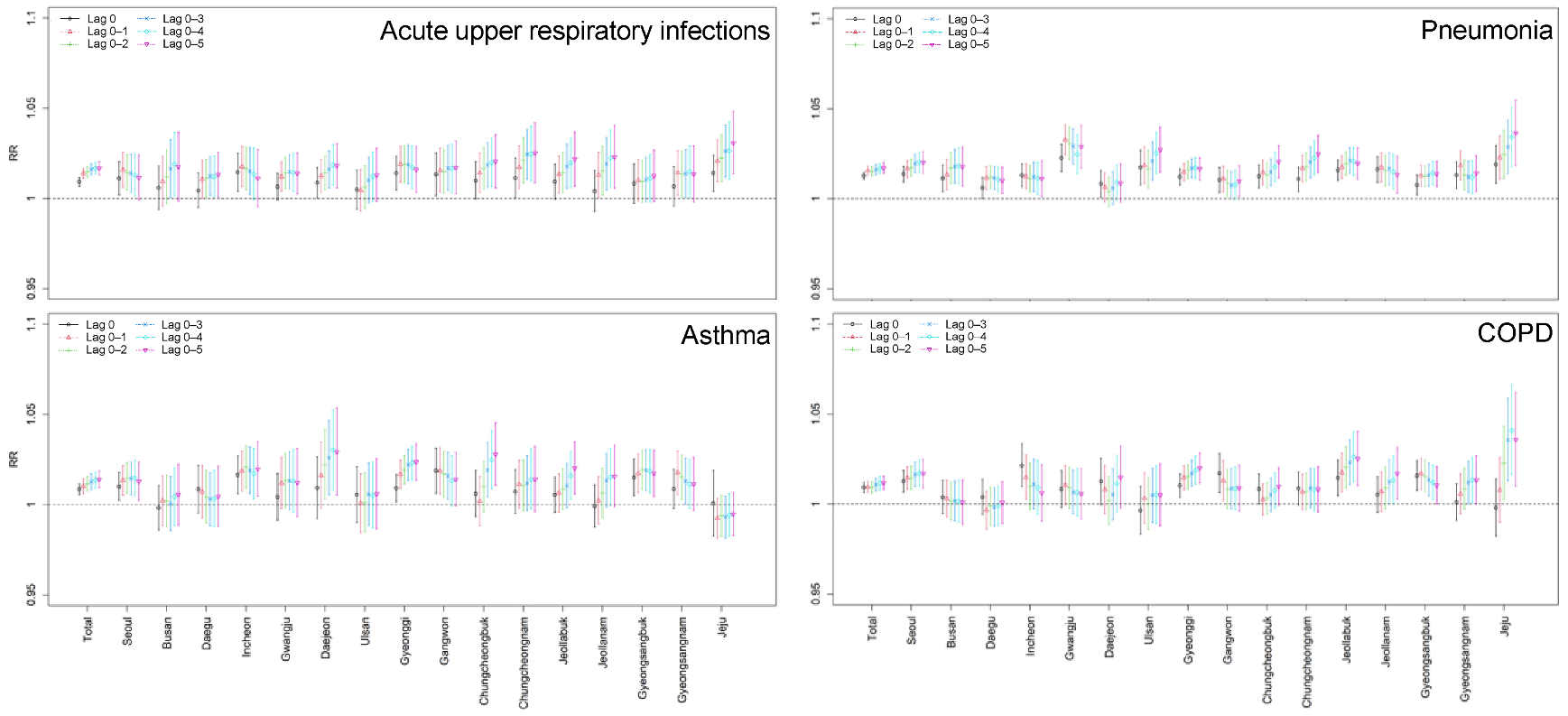


* COPD, chronic obstructive pulmonary disease; PM_10_, particulate matter ≤ 10 µm in diameter; RR, relative risk per 1°C increase in daily maximum temperature. Lag 0 represents same-day exposure, and lag 0–k represents the cumulative moving average of daily maximum temperature from day 0 to day k.

Supplementary Fig. 4 Relationship between daily maximum temperature and number of total respiratory diseases by lag days and regions, considering the second sensitivity analysis using the heat index instead of daily maximum temperature


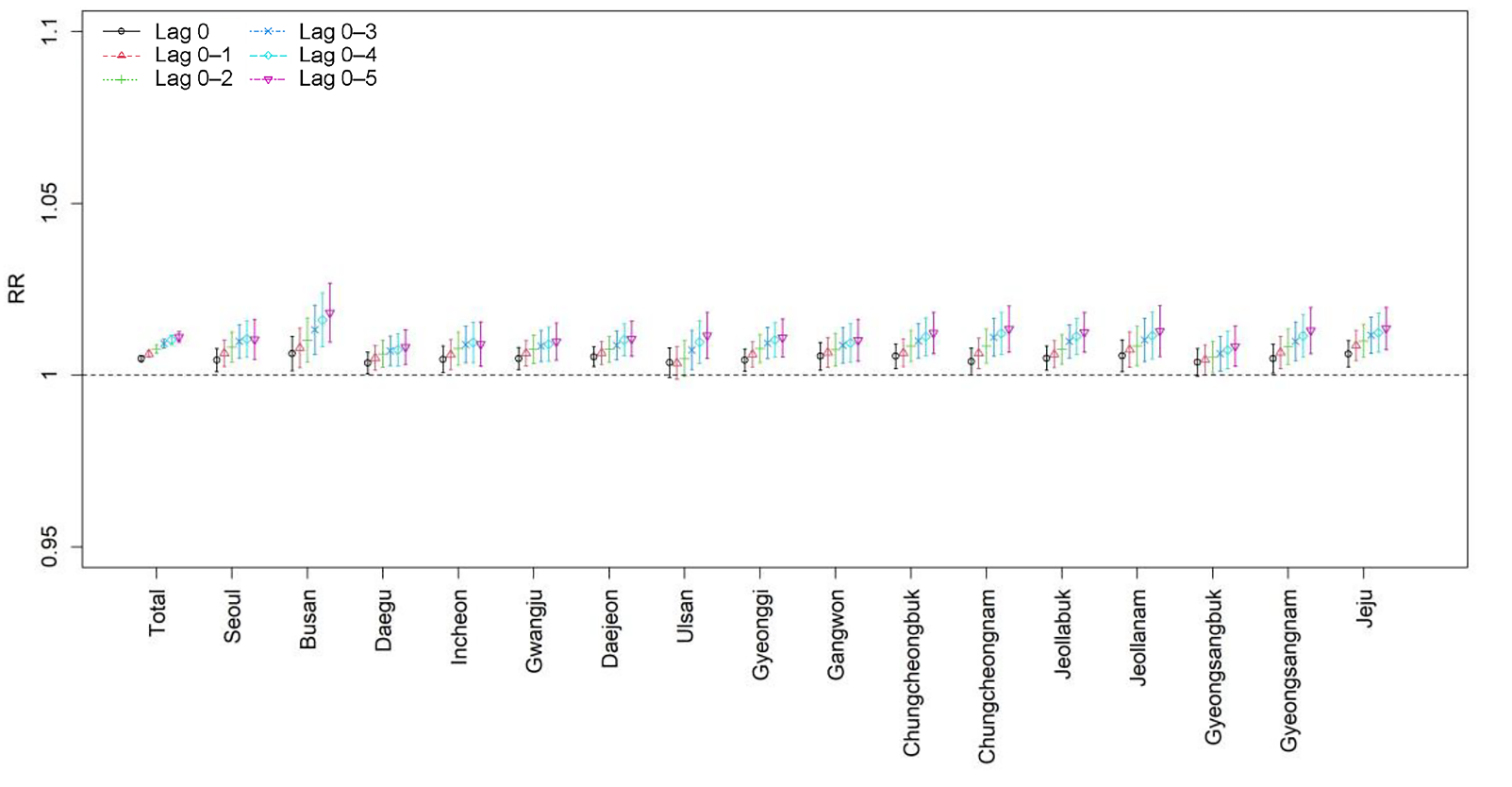


* RR, relative risk per 1°C increase in daily maximum temperature. Lag 0 represents same-day exposure, and lag 0–k represents the cumulative moving average of daily maximum temperature from day 0 to day k.

Supplementary Fig. 5 Relationship between daily maximum temperature and number of specific respiratory diseases (acute upper respiratory infections, pneumonia, asthma, and COPD) by lag days and regions, considering the second sensitivity analysis using heat index instead of daily maximum temperature


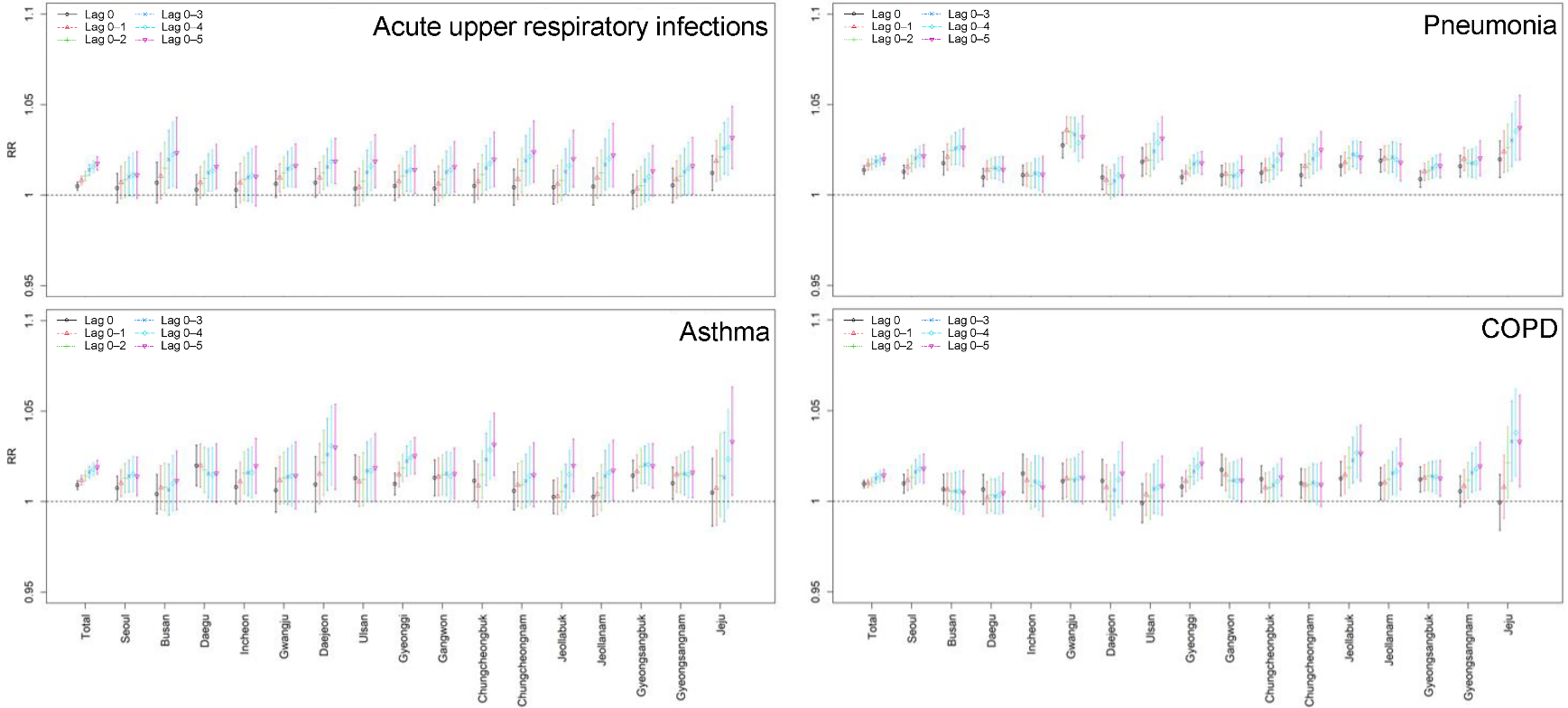


* COPD, chronic obstructive pulmonary disease; RR, relative risk per 1°C increase in daily maximum temperature. Lag 0 represents same-day exposure, and lag 0–k represents the cumulative moving average of daily maximum temperature from day 0 to day k.

Supplementary Fig. 6 Positive nonlinear relationship between daily maximum temperatures and total respiratory diseases across 16 different regions in South Korea at lag0, employing a time-series model that controlled for air pollutants (PM_10_, SO_2_, NO_2_, and O_3_) by including them as covariates in the first sensitivity analysis


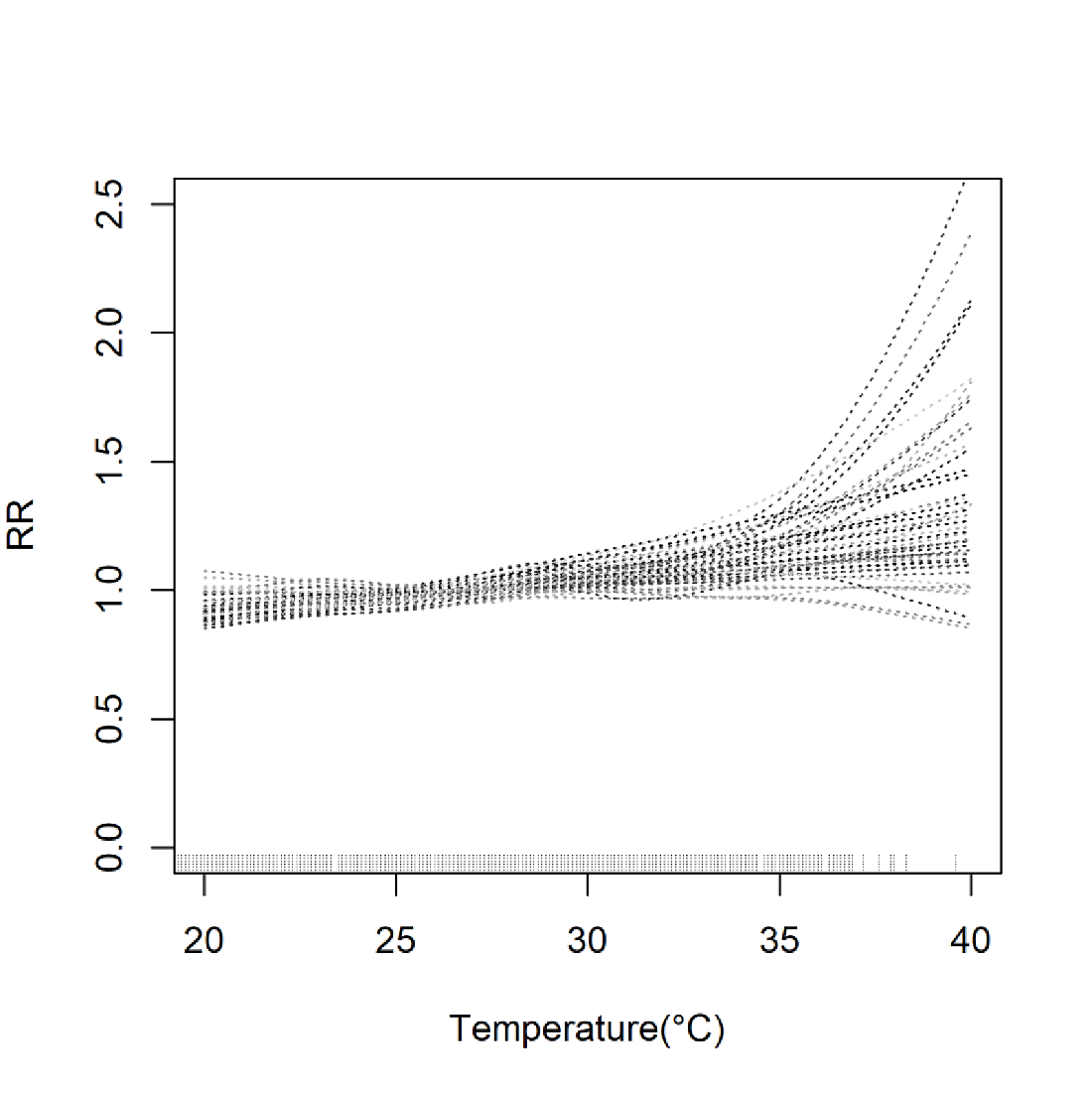


* PM_10_, particulate matter ≤10 µm in diameter; RR, relative risk per 1°C increase in daily maximum temperature.

* Each line in the figures represents one of the 16 regions, with the colour of the lines transitioning from black to lighter shades of grey according to the latitude of each region.

Supplementary Fig. 7 Positive nonlinear relationship observed between daily maximum temperatures and specific respiratory diseases (acute upper respiratory infections, pneumonia, asthma, and COPD) across 16 different regions in South Korea, after controlling for air pollutants (PM_10_, SO_2_, NO_2_, and O_3_) in the first sensitivity analysis


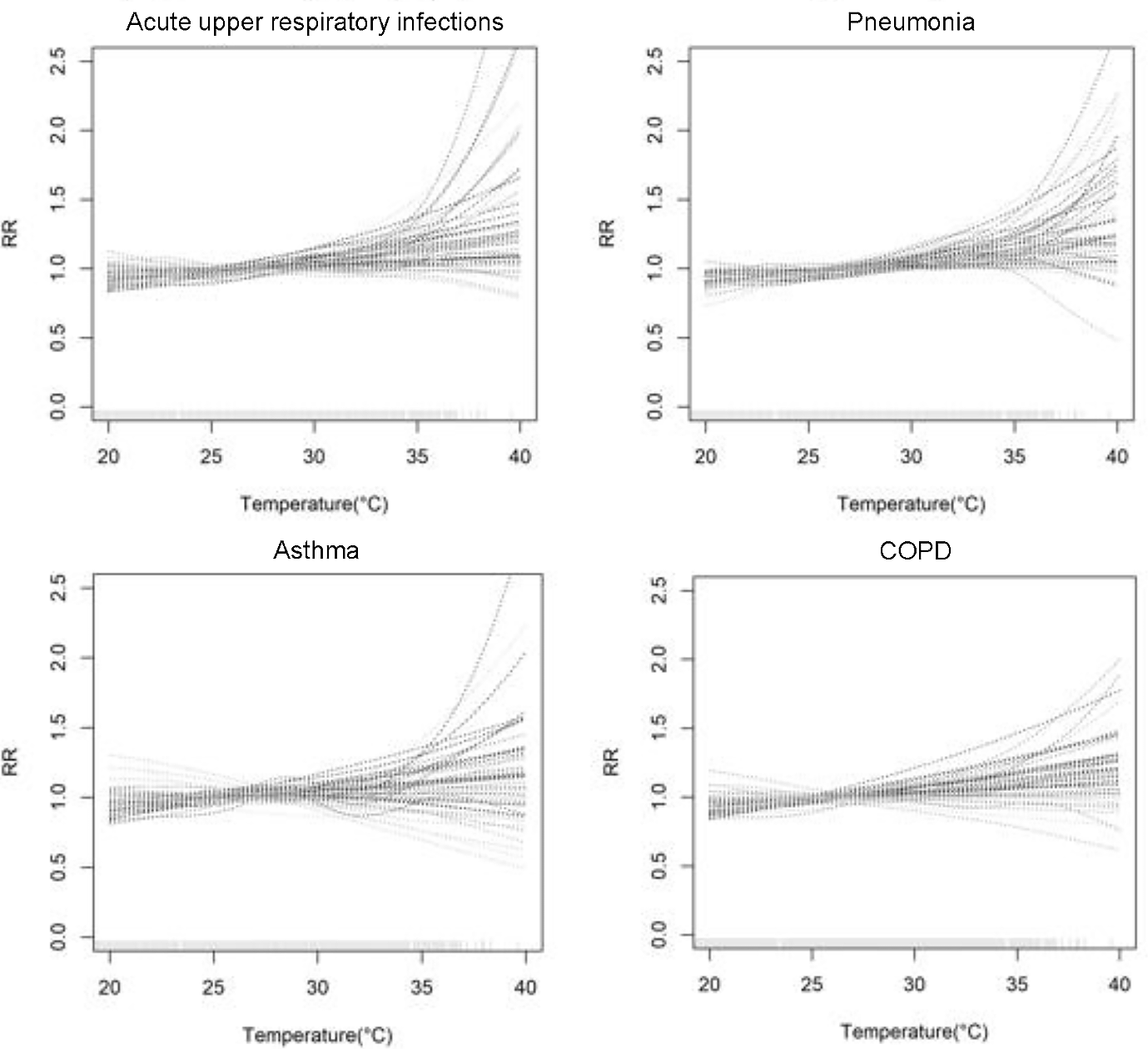


* COPD, chronic obstructive pulmonary disease; PM_10_, particulate matter ≤10 µm in diameter; RR, relative risk per 1°C increase in daily maximum temperature.

* Each line in the figures represents one of the 16 regions, with the colour of the lines transitioning from black to lighter shades of grey according to the latitude of each region.

Supplementary Fig. 8 Positive nonlinear relationship between daily maximum temperatures and total respiratory diseases across 16 different regions in South Korea at lag day 0, using the heat index instead of the daily maximum temperature in the second sensitivity analysis


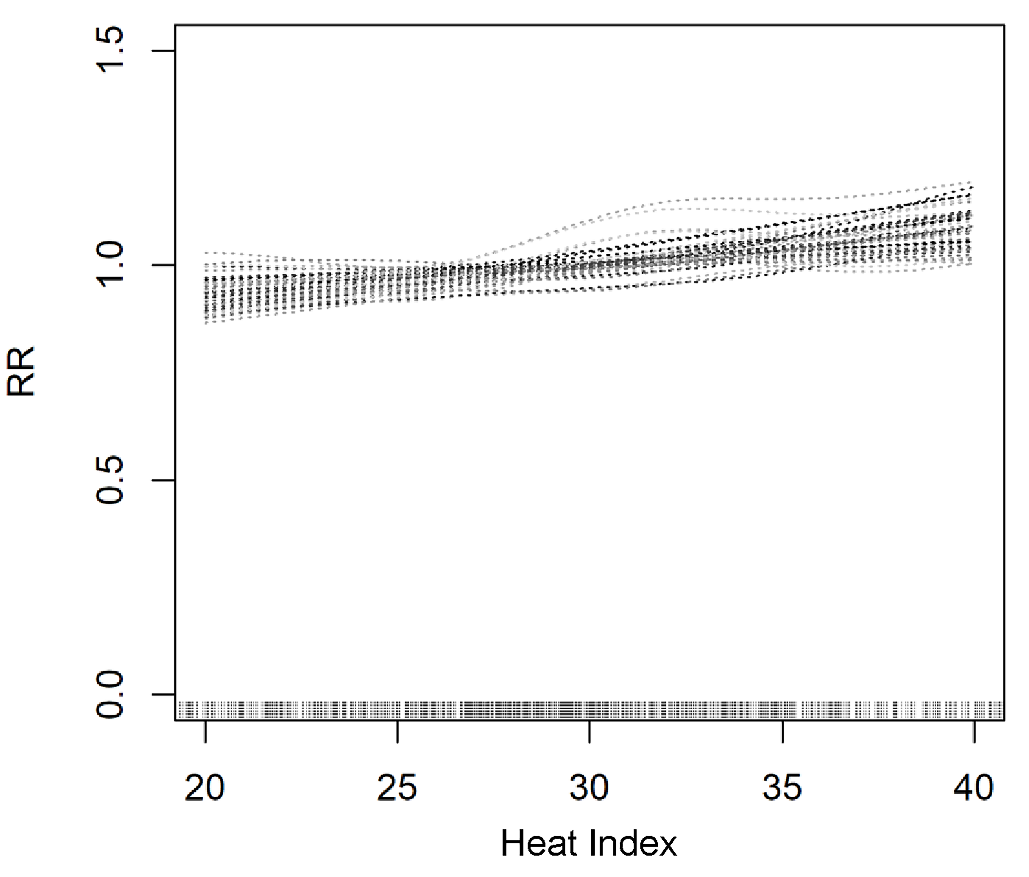


* RR, relative risk per 1°C increase in daily maximum temperature.

* Each line in the figures represents one of the 16 regions, with the colour of the lines transitioning from black to lighter shades of grey according to the latitude of each region.

Supplementary Fig. 9 Positive nonlinear relationship between daily maximum temperatures and specific respiratory diseases (acute upper respiratory infections, pneumonia, asthma, and COPD) across 16 different regions in South Korea at lag0, using the heat index instead of the daily maximum temperature in the second sensitivity analysis


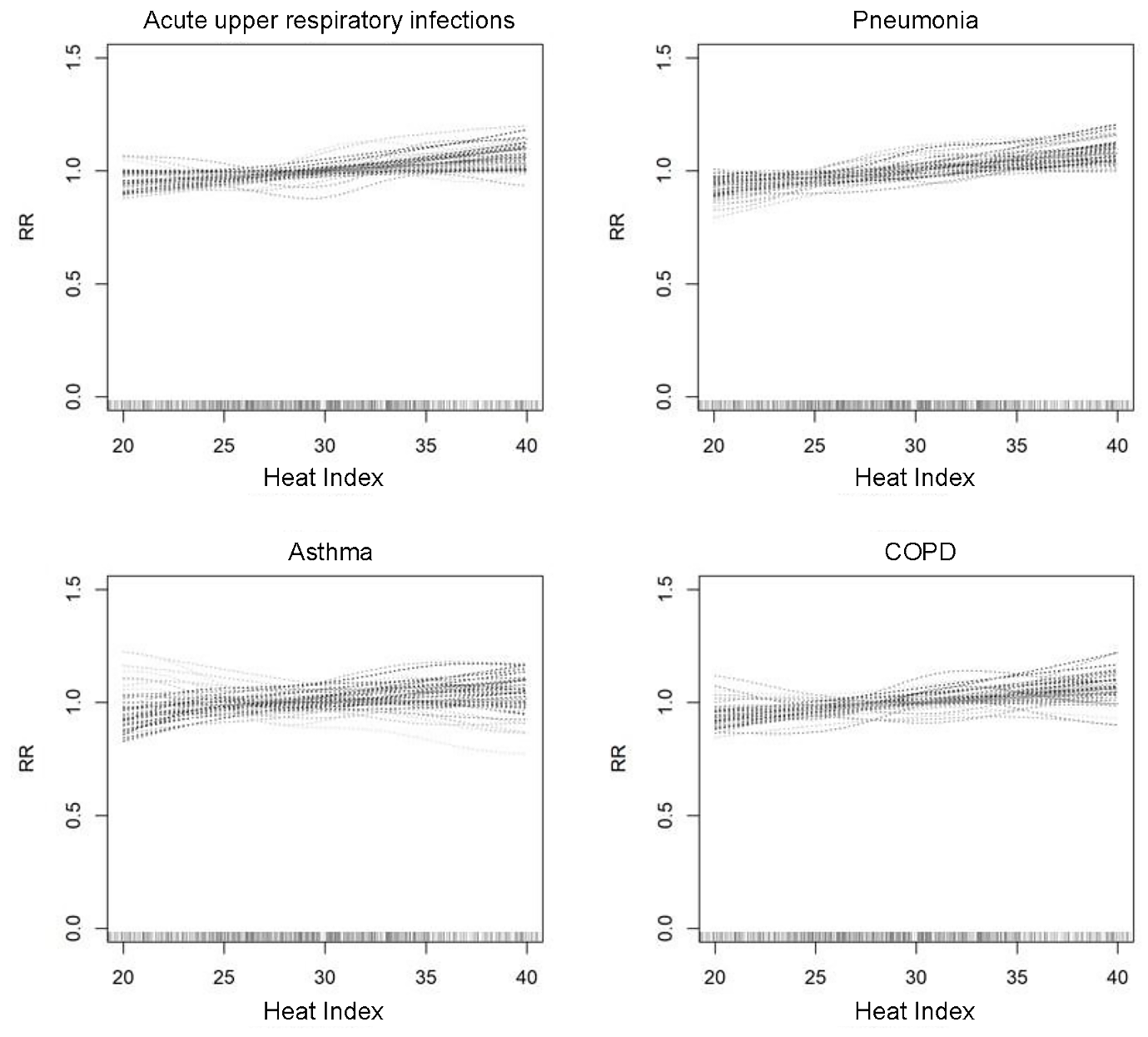


* COPD, chronic obstructive pulmonary disease; RR, relative risk per 1°C increase in daily maximum temperature.

* Each line in the figures represents one of the 16 regions, with the colour of the lines transitioning from black to lighter shades of grey according to the latitude of each region.

Supplementary Fig. 10 Subgroup analyses of the associations between daily maximum temperature and specific respiratory diseases (acute upper respiratory infections, pneumonia, asthma, and COPD) stratified by sex and age groups, controlling for air pollutants (PM_10_, SO_2_, NO_2_, and O_3_) in the first sensitivity analysis


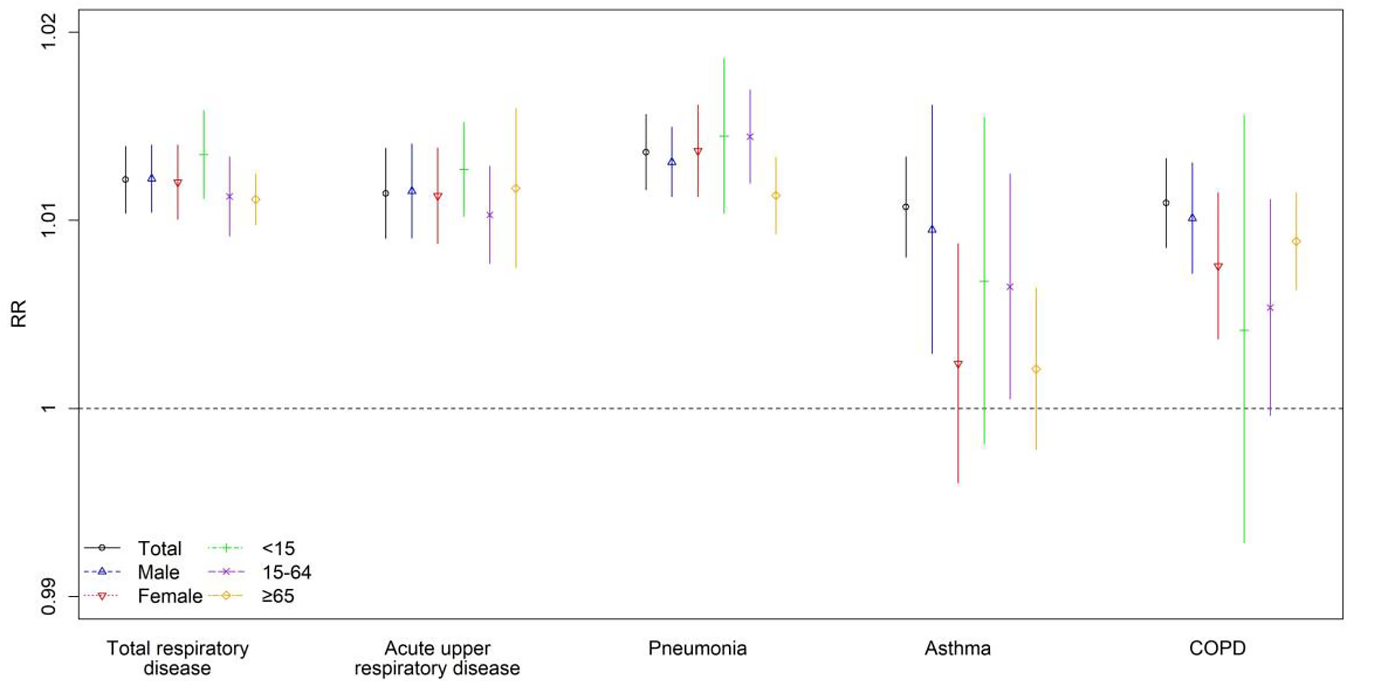


* COPD, chronic obstructive pulmonary disease; PM_10_, particulate matter ≤10 µm in diameter; RR, relative risk per 1°C increase in daily maximum temperature.

Supplementary Fig. 11 Subgroup analyses of the associations between daily maximum temperature and specific respiratory diseases (acute upper respiratory infections, pneumonia, asthma, and COPD) stratified by sex and age groups using the heat index instead of daily maximum temperature in the second sensitivity analysis


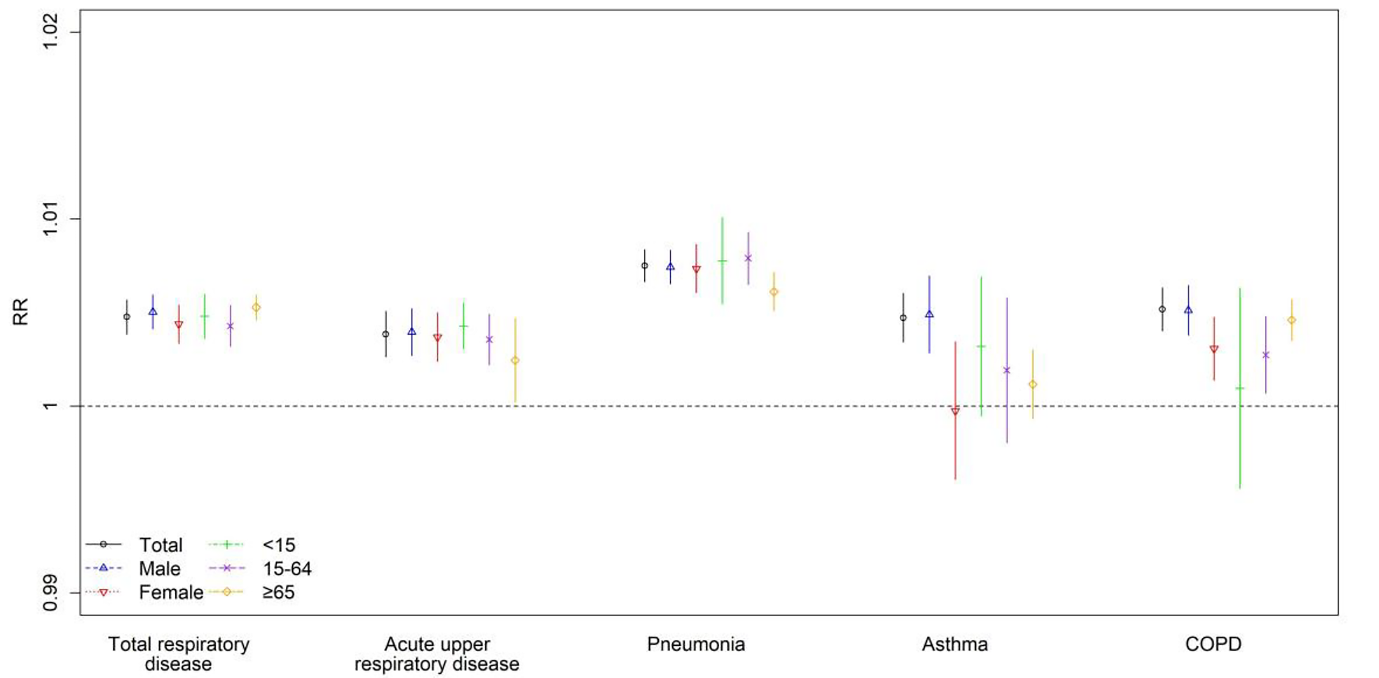


* COPD, chronic obstructive pulmonary disease; RR, relative risk per 1°C increase in daily maximum temperature.

**Supplementary Table 1.** City- and province-level population, emergency department visits, and respiratory diseases during the warm season

| Cities and provinces | Average  population (2014-2019) | Average daily number of emergency visit | | | | | | | | | | |
| --- | --- | --- | --- | --- | --- | --- | --- | --- | --- | --- | --- | --- |
|  |  | Total respiratory diseases | | | | | |  | Specific diseases | | | |
|  |  | Overall | Male | Female | <15 | 15-64 | ≥65 |  | Acute upper respiratory infections | Pneumonia | Asthma | COPD |
| Seoul Capital City | 9,772,570 | 527,791 | 286,709 | 241,082 | 231,945 | 190,614 | 105,232 |  | 301,937 | 119,519 | 23,104 | 38,278 |
| Busan City | 3,447,614 | 257,132 | 131,682 | 125,450 | 85,701 | 129,306 | 42,125 |  | 177,735 | 37,921 | 8,270 | 14,756 |
| Daegu City | 2,459,418 | 123,107 | 66,340 | 56,767 | 37,150 | 59,529 | 26,428 |  | 74,076 | 26,121 | 4,910 | 7,106 |
| Incheon City | 2,903,784 | 210,428 | 111,706 | 98,722 | 97,378 | 78,716 | 34,334 |  | 119,609 | 42,777 | 11,437 | 12,127 |
| Gwangju City | 1,458,434 | 178,099 | 91,609 | 86,490 | 61,400 | 99,261 | 17,438 |  | 136,680 | 20,815 | 4,823 | 7,907 |
| Daejeon City | 1,499,266 | 77,447 | 42,059 | 35,388 | 32,161 | 30,155 | 15,131 |  | 45,676 | 17,576 | 3,148 | 3,503 |
| Ulsan City | 1,157,785 | 96,460 | 51,130 | 45,330 | 31,213 | 53,102 | 12,145 |  | 67,124 | 11,041 | 3,287 | 7,146 |
| Gyeonggi-do | 12,604,945 | 802,578 | 434,912 | 367,666 | 382,832 | 288,723 | 131,023 |  | 486,002 | 158,462 | 35,318 | 44,192 |
| Gangwon-do | 1,532,960 | 123,750 | 66,382 | 57,368 | 42,527 | 46,442 | 34,781 |  | 71,269 | 28,567 | 6,525 | 9,094 |
| Chungcheongbuk-do | 1,578,694 | 101,895 | 55,374 | 46,521 | 31,513 | 41,757 | 28,625 |  | 56,871 | 24,433 | 5,408 | 8,761 |
| Chungcheongnam-do | 2,079,976 | 180,119 | 99,612 | 80,507 | 71,346 | 67,835 | 40,938 |  | 110,295 | 36,492 | 8,554 | 12,629 |
| Jeollabuk-do | 1,845,059 | 153,969 | 84,323 | 69,646 | 35,572 | 73,623 | 44,774 |  | 88,443 | 34,060 | 9,069 | 13,367 |
| Jeollanam-do | 1,884,681 | 251,935 | 134,086 | 117,849 | 62,373 | 130,733 | 58,829 |  | 175,670 | 34,728 | 12,509 | 17,822 |
| Gyeongsangbuk-do | 2,674,787 | 224,976 | 121,282 | 103,694 | 71,207 | 96,446 | 57,323 |  | 137,999 | 44,791 | 10,849 | 19,088 |
| Gyeongsangnam-do | 3,344,603 | 319,845 | 169,116 | 150,729 | 108,497 | 165,083 | 46,265 |  | 236,040 | 37,472 | 10,823 | 16,926 |
| Jeju-do | 633,284 | 66,192 | 34,922 | 31,270 | 31,915 | 21,074 | 13,203 |  | 37,116 | 15,967 | 3,041 | 4,884 |

Abbreviations: COPD, chronic obstructive pulmonary disease.
